# The role of regulatory T cells in infant HIV pathogenesis: therapeutic strategies

**DOI:** 10.1097/MS9.0000000000003631

**Published:** 2025-07-22

**Authors:** Emmanuel Ifeanyi Obeagu, Raajasiri Iyengar

**Affiliations:** a Department of Biomedical and Laboratory Science, Africa University, Zimbabwe; b Department of GA, India

**Keywords:** immune activation, immunoregulation, infant HIV, therapeutic targeting, viral persistence

## Abstract

Regulatory T cells (Tregs) are pivotal in maintaining immune homeostasis by suppressing excessive immune responses, thereby preventing immunopathology. In the context of infant human immunodeficiency virus (HIV) infection, Tregs exhibit a dualistic role: while they mitigate immune activation, they may also impede effective antiviral immunity, facilitating viral persistence. Recent studies have illuminated the nuanced involvement of Tregs in infant HIV pathogenesis. For instance, research has demonstrated that HIV-exposed uninfected infants exhibit lower frequencies of peripheral blood Tregs at birth compared to unexposed infants, leading to a delayed expansion of these cells over the first 36 weeks of life. This disruption in Treg development is associated with gut epithelial damage, suggesting that compromised mucosal integrity may influence Treg dynamics in early life. Tregs influence HIV pathogenesis in infants through several mechanisms. They suppress the activation and proliferation of effector T cells, including HIV-specific CD8+ cytotoxic T lymphocytes, which are crucial for controlling viral replication. This suppression can lead to inadequate immune responses against HIV, allowing the virus to persist and replicate. Additionally, Tregs can modulate the function of dendritic cells, leading to suboptimal antigen presentation and further dampening the adaptive immune response. Moreover, an imbalance between Tregs and Th17 cells, another subset of CD4+ T cells involved in mucosal immunity, has been observed in HIV-infected individuals. The loss of Th17 cells, coupled with an increase in Tregs, can compromise mucosal barriers, facilitating microbial translocation and chronic immune activation, which are hallmarks of HIV disease progression.

## Introduction

Regulatory T cells (Tregs) are a specialized subset of CD4+ T cells characterized by the expression of the transcription factor FoxP3. They play a pivotal role in maintaining immune homeostasis by suppressing excessive immune responses and preventing autoimmunity. In the context of HIV infection, Tregs have garnered significant attention due to their dualistic role: while they can mitigate immune activation, they may also suppress HIV-specific immune responses, potentially facilitating viral persistence[1]. In HIV-infected adults, studies have shown that Treg frequencies within the CD4+ T cell compartment often increase as the disease progresses. This relative increase is primarily attributed to the decline in overall CD4+ T cell counts rather than a true expansion of Tregs. Interestingly, while the relative frequency of Tregs rises, their absolute numbers tend to decrease, correlating with heightened immune activation and elevated plasma viral loads. This phenomenon underscores the complex interplay between Tregs and the broader immune landscape in HIV infection[2]. Phenotypically, Tregs in HIV-infected individuals exhibit distinct characteristics. For instance, during primary HIV infection, Tregs display elevated expression levels of FoxP3, ICOS, and CTLA-4. Notably, CTLA-4 expression is markedly increased across all Treg subsets, both at the initial diagnosis and 6 months thereafter. These phenotypic alterations may influence the suppressive capacity of Tregs, thereby affecting the overall immune response to HIV[3]. The balance between Tregs and Th17 cells, another subset of CD4+ T cells, is particularly pertinent in HIV infection. Th17 cells are integral to mucosal immunity, and their depletion is a hallmark of HIV disease progression. In healthy individuals, the Th17/Treg ratio typically ranges from 1.0 to 1.2. However, in HIV-infected patients, this ratio decreases significantly, ranging from 0.75 to 0.2. This imbalance can compromise mucosal barriers, leading to microbial translocation and chronic immune activation, which are central to HIV pathogenesis[4].

**HIGHLIGHTS**

- Immunoregulation: Regulatory T cells (Tregs) play a crucial role in modulating the immune response, maintaining immune homeostasis, and preventing autoimmunity, which is essential in the context of HIV infection in infants.
- Viral persistence: Tregs can contribute to viral persistence.
- Immune activation: In HIV-infected infants, Tregs help regulate the heightened immune activation.
- HIV reservoirs: Tregs are implicated in the maintenance and formation of HIV reservoirs.
- Therapeutic targeting: Understanding the role of Tregs in infant HIV pathogenesis offers potential therapeutic opportunities.

In pediatric populations, the dynamics of Tregs in the context of HIV infection present unique challenges. Neonates naturally have a higher frequency of Tregs compared to adults, reflecting an immune system skewed towards tolerance. This predisposition is crucial for preventing overactive immune responses during early development. However, in HIV-infected infants, this heightened Treg presence may inadvertently suppress effective antiviral responses, facilitating viral persistence[5]. Recent studies have highlighted alterations in Treg subsets during primary HIV infection. For example, a study observed that while the overall frequency of Tregs remained comparable between patients and healthy donors, Tregs from HIV-infected individuals exhibited elevated expression of markers such as FoxP3, ICOS, and CTLA-4. These phenotypic changes suggest an enhanced suppressive capacity, which could dampen HIV-specific immune responses, thereby contributing to viral persistence[6]. Furthermore, the tissue distribution of Tregs plays a significant role in HIV pathogenesis. Lymphoid tissues, which serve as primary sites for HIV replication, have been found to harbor higher frequencies of Tregs compared to peripheral blood. This accumulation in lymphoid tissues may suppress local HIV-specific cytotoxic T lymphocyte (CTL) responses, hindering effective viral control. Notably, even in advanced stages of HIV disease, Tregs in lymphoid tissues retain their suppressive function, emphasizing their potential role in sustaining viral reservoirs[7]. The functional capacity of Tregs in HIV-infected individuals is also of paramount importance. Despite the chronic immune activation characteristic of HIV infection, Tregs maintain their suppressive functions. This persistent activity can inhibit HIV-specific T cell responses, potentially facilitating ongoing viral replication and disease progression^[8,9]^.

## Aim

This review aims to comprehensively explore the current understanding of Tregs in pediatric HIV infection, focusing on their role in immune regulation, disease pathogenesis, clinical implications, and potential therapeutic interventions.

## Regulatory T cells

Tregs are a specialized subset of CD4+ T cells characterized by the expression of the transcription factor FoxP3. They play a pivotal role in maintaining immune homeostasis by suppressing excessive immune responses and preventing autoimmunity. In the context of HIV infection, Tregs exhibit a dualistic role: while they can mitigate immune activation, they may also suppress HIV-specific immune responses, potentially facilitating viral persistence. Recent research has shed light on the complex role of Tregs in HIV infection. Studies have shown that HIV-1 can directly infect Tregs, disturbing their phenotype and suppressive capacity via different mechanisms, including FoxP3 and CD25 downregulation, and impairment of suppressive capacity. These alterations can disrupt the delicate balance of immune regulation, leading to either uncontrolled immune activation or inadequate antiviral responses^[9,10]^. Treg plasticity, or the ability of Tregs to adapt and differentiate into other T cell subsets, has significant implications in HIV infection. This adaptability allows Tregs to respond to varying immunological environments, but in the context of HIV, it may lead to the loss of regulatory functions and contribute to disease progression. For instance, the conversion of Tregs into pro-inflammatory Th17 cells under certain conditions can exacerbate immune activation, a hallmark of HIV pathogenesis[11].

HIV’s impact on Tregs is multifaceted. The virus can directly infect Tregs, leading to functional impairments. Additionally, HIV infection is associated with immune activation, which can result in the depletion of Tregs. A study involving HIV-positive Ugandan volunteers found that Treg number is strongly correlated with both CD4+ and CD8+ T cell activation[11]. In multivariate modeling, this relationship between Treg depletion and CD4+ T cell activation was stronger than any other clinical factor examined, including viral load and absolute CD4 count. This suggests that Tregs are a major contributor to the immune activation observed during chronic HIV infection[12]. The functional mechanisms of Tregs in modulating immune activation during HIV infection involve various pathways. These include the secretion of cytokines like IL-10 and TGF-β, expression of ectoenzymes such as CD39 and CD73, and modulation of intracellular molecules like cyclic adenosine monophosphate. HIV infection can disrupt these regulatory mechanisms, leading to immune dysregulation. For example, alterations in the CD39/CD73 axis can impair the suppressive function of Tregs, contributing to heightened immune activation[13]. The balance between Tregs and effector T cells is crucial in HIV infection. While Tregs help control immune activation, they can also suppress HIV-specific effector responses, hindering the body’s ability to combat the virus effectively. *Ex vivo* depletion of Tregs from peripheral blood mononuclear cells or lymphoid cell suspensions has been shown to enhance T cell responses to HIV or SIV antigens, suggesting that Tregs dampen HIV/SIV responses. This highlights the complex role of Tregs in HIV infection, where they can have both beneficial and detrimental effects[14].

## Role of Tregs in infant immune development

Tregs are a specialized subset of CD4+ T cells that play a crucial role in maintaining immune homeostasis by suppressing excessive immune responses and preventing autoimmunity. In infants, Tregs are instrumental in guiding the maturation of the immune system, ensuring a balanced response to environmental antigens and establishing tolerance to self-antigens. Recent research has illuminated the intricate interplay between Tregs and the gut microbiota, highlighting their collective influence on infant immune development[15].

## Tregs and infant immune development

During early life, the immune system undergoes rapid maturation, transitioning from a naive state to one capable of distinguishing between harmful pathogens and benign antigens. Tregs are central to this process, modulating immune responses to both self and foreign antigens. Recent studies have demonstrated that the composition of the gut microbiota significantly influences Treg development in infants. For instance, specific bacterial metabolites, such as short-chain fatty acids (SCFAs) like butyrate, have been shown to promote the differentiation and function of Tregs, thereby contributing to immune regulation[16].

## Mechanisms of Treg-microbiota interactions

The gut microbiota interacts with Tregs through several mechanisms:

1. Metabolite production: Certain gut bacteria produce SCFAs, such as butyrate, which promote Treg differentiation and function.
2. Antigen presentation: Microbial antigens can be presented by dendritic cells, leading to the induction of Tregs that are specific to these antigens.
3. Toll-like receptor (TLR) signaling: Microbial components can engage TLRs on immune cells, influencing Treg development and activity.

These interactions underscore the symbiotic relationship between the microbiota and the immune system, where microbial signals are essential for the proper development and function of Tregs[17].

## Impact of dysbiosis on Treg function

Dysbiosis, defined as an imbalance in the microbial community, can disrupt Treg function and overall immune regulation in infants. Factors such as antibiotic use, cesarean delivery, and formula feeding can contribute to dysbiosis. This imbalance may lead to reduced SCFA production, impairing Treg induction and function, and potentially resulting in heightened susceptibility to infections and inflammatory conditions. Moreover, dysbiosis has been associated with a decreased production of butyrate, a key SCFA involved in Treg differentiation, further compromising immune tolerance[18].

## Treg dysregulation in infants exposed to maternal HIV

Infants born to HIV-positive mothers, termed HIV-exposed uninfected (HEU) infants, present a unique context to study Treg dysregulation. Maternal HIV infection can influence the infant’s gut microbiota, subsequently affecting Treg development and immune regulation. Studies have shown that alterations in the gut microbiota composition in HEU infants are linked to immune dysregulation, highlighting the impact of microbial composition on Treg function and overall immune health in infants[19].

## Dysbiosis and immune dysregulation in HEU infants

A case study of HEU infants demonstrated that alterations in the gut microbiota composition were linked to immune dysregulation. These infants exhibited an increased abundance of certain bacterial species, which correlated with elevated expression of inhibitory receptors on Tregs. This upregulation may contribute to impaired immune responses, highlighting the impact of microbial composition on Treg function and overall immune health in infants[19].

## Impact of HIV on Treg dynamics

Tregs are a specialized subset of CD4+ T cells that play a pivotal role in maintaining immune tolerance and preventing autoimmunity. In the context of HIV infection, Tregs have garnered significant attention due to their complex involvement in disease progression and immune system dynamics[20].

## Treg accumulation in lymphoid tissues

One of the notable observations in HIV-infected individuals is the accumulation of Tregs in lymphoid tissues. This accumulation has been associated with disease progression. A study published in *Blood* reported increased numbers of FOXP3+ T cells, a marker for Tregs, in lymphoid tissues during progressive HIV infection. This increase was not correlated with immune activation, suggesting a unique mechanism of Treg accumulation in these tissues. The study also found that a high perforin/FOXP3 ratio was associated with nonprogressive disease, indicating that the balance between effector immune responses and Treg-mediated regulation is crucial for controlling HIV replication^[6,21]^. Table 1 shows the role of Tregs in infant HIV pathogenesis (provided by the author).

**Table 1:** Summarizing the role of regulatory T cells (Tregs) in infant HIV pathogenesis

Aspect	Description	Research/findings	Implications
Treg frequency in infants	Tregs play a critical role in maintaining immune tolerance and regulating immune responses in infants.	Studies indicate that HIV-infected infants often show altered Treg frequencies compared to healthy controls. Treg frequency may be increased due to persistent immune activation.	Altered Treg frequencies could contribute to immune dysfunction, influencing HIV progression and resistance to antiviral treatments.
Treg function in HIV	Tregs help suppress immune responses but can impair the development of effective HIV-specific immunity.	HIV infection in infants is associated with Treg-mediated suppression of immune responses, leading to insufficient control over viral replication.	Treg dysfunction or overactivation can hinder the development of strong adaptive immune responses, exacerbating HIV pathogenesis.
Treg subsets in infants	Different subsets of Tregs, such as Th17/Tregs, may play unique roles in infant HIV pathogenesis.	Studies show increased proportions of Th17 cells in HIV-infected infants, which may affect Treg plasticity and alter immune responses.	The presence of Th17 cells might impact Treg functionality, leading to dysregulation of both innate and adaptive immune responses in infants
Impact of maternal HIV	Maternal HIV can influence Treg development and function in infants, potentially leading to dysregulation.	Maternal HIV exposure during pregnancy has been shown to affect the neonatal immune system, including Treg induction and immune tolerance mechanisms.	Dysregulation of Tregs in infants born to HIV-infected mothers could result in higher susceptibility to infections and slower immune maturation.
Dysbiosis and Treg impact	Dysbiosis (imbalance in the gut microbiota) influences Treg differentiation and function.	Recent studies suggest that HIV-infected infants exhibit altered microbiota profiles, which could impact Treg-mediated immune regulation and lead to immune dysfunction.	Dysbiosis may impair Treg differentiation, potentially leading to poor regulation of immune responses and increased inflammation in HIV-infected infants.
Treg plasticity	Tregs are highly adaptable and can switch phenotypes, affecting their role in HIV progression.	Research shows that HIV infection induces changes in Treg plasticity, allowing them to become less suppressive and potentially contributing to inflammation and immune escape.	Treg plasticity may contribute to persistent inflammation and viral replication by reducing Treg suppressive functions, promoting chronic infection in infants.
HIV reservoirs and Tregs	Tregs may contribute to the persistence of viral reservoirs by suppressing immune responses.	HIV reservoirs in infants are thought to persist in part due to the suppressive functions of Tregs, which limit the effectiveness of anti-HIV immune responses.	Modulating Treg function may help eliminate viral reservoirs and improve immune control of HIV, reducing long-term HIV-related morbidity in infants.
Impact of Treg dysregulation	Treg dysregulation can lead to immune hyperactivation or immune tolerance failure.	Studies in HIV-infected infants suggest that Treg dysfunction could lead to increased inflammation and immune activation, contributing to disease progression.	Proper regulation of Tregs is essential to balance immune tolerance and activation, ensuring that immune responses do not damage the host or fail to control the virus

## Mechanisms of Treg expansion in HIV infection

The expansion of Tregs during HIV infection can be attributed to several factors:

1. Direct viral interaction: HIV can directly interact with Tregs, promoting their survival. This interaction is mediated through the binding of the HIV envelope protein gp120 to the CD4 receptor on Tregs, leading to enhanced Treg survival and accumulation in lymphoid tissues[15].
2. Antigenic stimulation: Chronic exposure to HIV antigens can drive the expansion of Tregs. This persistent antigenic stimulation may lead to the generation of adaptive Tregs from mature T-cell populations, contributing to their increased numbers during HIV infection[15].
3. Cytokine environment: The cytokine milieu during HIV infection, characterized by elevated levels of cytokines such as IL-10 and TGF-β, can facilitate the differentiation and expansion of Tregs. These cytokines create an environment conducive to Treg proliferation, further contributing to their accumulation[15].

## Implications of Treg expansion on HIV disease progression

The accumulation and expansion of Tregs in HIV-infected individuals have significant implications for disease progression:

- Suppression of HIV-specific immune responses: Tregs can suppress the function of HIV-specific CD4+ and CD8+ T cells, leading to diminished immune responses against the virus. This suppression hampers the body’s ability to control HIV replication effectively[22].
- Th17/Treg imbalance and mucosal immunity: HIV infection is associated with a depletion of Th17 cells, which are crucial for maintaining mucosal immunity. The imbalance between Th17 cells and Tregs can result in a breakdown of mucosal barriers, increased microbial translocation, and heightened systemic immune activation, all of which contribute to disease progression[22].

## Tregs as a reservoir for HIV

Emerging evidence suggests that Tregs may serve as a reservoir for latent HIV:

- Persistence of proviruses: Studies have shown higher frequencies of inducible, intact HIV proviruses in Tregs compared to other CD4+ T cell subsets. This finding indicates that Tregs can harbor latent HIV, posing a challenge for eradication efforts[23].
- Resistance to apoptosis: Tregs are known to be long-lived and resistant to apoptosis. These characteristics make them a stable reservoir for HIV, allowing the virus to persist despite antiretroviral therapy[23].

## Clinical implications and disease progression

Tregs are pivotal in maintaining immune homeostasis by suppressing excessive immune responses and preventing autoimmunity. In the context of HIV infection, Tregs have a dualistic role, influencing both disease progression and the efficacy of therapeutic interventions[24].

## Tregs and HIV disease progression

During HIV infection, Tregs accumulate in lymphoid tissues, a phenomenon associated with disease progression. This accumulation can suppress HIV-specific immune responses, thereby facilitating viral persistence. The immunosuppressive function of Tregs, while preventing excessive immune activation, may inadvertently impair the body’s ability to mount effective responses against HIV, leading to unchecked viral replication and progression to Acquired Immunodeficiency Syndrome[25]. Recent studies have highlighted that Tregs can serve as reservoirs for latent HIV. Their resistance to apoptosis and long lifespan make them suitable hosts for the virus, complicating eradication efforts. This reservoir potential underscores the challenges in achieving complete viral clearance, even with effective antiretroviral therapy (ART)^[25,26]^.

## TIGIT expression and immune exhaustion

T-cell immunoreceptor with Ig and ITIM domains (TIGIT) is an inhibitory receptor expressed on T cells, including Tregs. In HIV-infected individuals, there is an expansion of TIGIT-expressing CD8+ T cells, which correlates with disease progression. These cells often co-express Programmed Death Protein 1 (PD-1), another inhibitory receptor, and exhibit features of T-cell exhaustion – a state of dysfunction resulting from chronic antigen exposure. The co-expression of TIGIT and PD-1 marks a subset of T cells with diminished effector functions, contributing to inadequate viral control[27].

## Therapeutic implications

Understanding the role of Tregs and inhibitory receptors like TIGIT in HIV infection has significant clinical implications:

- Immune checkpoint blockade: Targeting inhibitory pathways involving TIGIT and PD-1 has emerged as a potential strategy to rejuvenate exhausted T cells. Blockade of these receptors can restore the functionality of HIV-specific T cells, enhancing their capacity to eliminate infected cells. This approach is being explored to improve immune responses in HIV-infected individuals, particularly those with suboptimal recovery despite ART[28].
- Treg modulation: Selective depletion or functional modulation of Tregs has been investigated to enhance immune activation against HIV. However, such strategies must be approached cautiously to avoid triggering autoimmunity or excessive immune activation, which could lead to detrimental inflammation[28].
- Targeting latent reservoirs: Since Tregs can harbor latent HIV, strategies aiming to purge these reservoirs are critical for achieving a functional cure. This includes “shock and kill” approaches, where latent viruses are reactivated (“shock”) and subsequently eliminated by the immune system or therapeutic agents (“kill”). Understanding the mechanisms of latency within Tregs is essential for developing effective eradication strategies[28].

## Therapeutic strategies targeting Tregs

Tregs play a pivotal role in maintaining immune homeostasis, and their manipulation has emerged as a potential therapeutic strategy in HIV infection. Recent studies have explored various approaches to modulate Treg activity, aiming to enhance anti-HIV immune responses^[29,30]^.

## Targeting CD25 for Treg depletion

CD25, the alpha chain of the IL-2 receptor, is constitutively expressed on Tregs and serves as a target for depletion strategies. Denileukin diftitox (Ontak) combines IL-2 with diphtheria toxin, binding to CD25+ cells and inducing cell death. In studies with humanized mouse models, Ontak administration led to reduced Treg levels and increased immune activation markers, suggesting potential in reducing viral reservoirs^[31,32]^.

## PD-1/PD-L1 pathway modulation

The PD-1/PD-L1 pathway negatively regulates T-cell responses. Blocking this interaction has shown promise in enhancing HIV-specific CD8+ T-cell responses. Studies indicate that PD-1 blockade restores the proliferative capacity of these cells, potentially improving immune control over HIV[33].

## IDO activity modulation

Indoleamine 2,3-dioxygenase (IDO) influences the Th17/Treg balance. Inhibiting IDO with agents like 1-methyl-d-tryptophan has been associated with enhanced HIV-specific cytotoxic T-lymphocyte responses, leading to the elimination of HIV-infected macrophages. However, combination therapies targeting both CTLA-4 and IDO have resulted in adverse effects, including fulminant diabetes, highlighting the need for cautious approach[34].

## Clinical trial insights

Clinical trials have provided insights into Treg modulation:

- A study involving recombinant IL-2 and IL-7 in HIV-infected adults observed immunomodulatory effects on Tregs, suggesting potential for enhancing immune responses[35].
- The use of Daclizumab, a monoclonal antibody targeting CD25, has shown promise in depleting Tregs and inducing remission in conditions like adult T-cell leukemia. However, its role in HIV therapy requires further investigation[35].

## Considerations and future directions

While Treg-targeted therapies offer potential, challenges remain:

- Safety concerns: Strategies like Treg depletion may lead to increased immune activation, posing risks of hyperimmune responses or autoimmunity[36].
- Timing and patient selection: Identifying optimal treatment windows and suitable patient populations is crucial to balance therapeutic benefits and risks[36].
- Long-term efficacy: Assessing the durability of responses and potential long-term effects of Treg-modulating therapies is essential^[37,38]^.

## Conclusion

The manipulation of Tregs holds significant potential for advancing HIV treatment strategies by restoring immune function and improving control over viral replication. However, the dynamic role of Tregs in both immune suppression and viral persistence presents challenges that must be carefully navigated in therapeutic applications. Recent studies have provided important insights into Treg frequencies and their impact on disease progression. For instance, a study published in *Frontiers in Immunology* demonstrated that HIV infection leads to an expansion of Tregs, particularly in the lymphoid tissues, which is associated with a higher viral load and impaired HIV-specific immune responses. These findings underscore the need for therapies that can either reduce Treg accumulation or modulate their function to promote more robust immune responses. Treg-targeting strategies have shown promise in preclinical and clinical settings, as evidenced by several recent trials. In a phase 1 clinical trial of the anti-CD25 monoclonal antibody Daclizumab, which targets Tregs, researchers observed a significant reduction in Treg levels and enhanced activation of HIV-specific CD8+ T cells, leading to improved control of viral replication in a small subset of participants. Furthermore, the use of recombinant IL-2 and IL-7 in HIV-infected individuals was associated with modulation of Treg activity, resulting in improved immune reconstitution and better overall immune responses.

## Data Availability

Not applicable as this is a narrative review.

## Research registration unique identifying number (UIN)

Not applicable as this is a narrative review.
